# Sensory Circuit Remodeling and Movement Recovery After Spinal Cord Injury

**DOI:** 10.3389/fnins.2021.787690

**Published:** 2021-12-08

**Authors:** Yunuen Moreno-López, Edmund R. Hollis

**Affiliations:** ^1^Burke Neurological Institute, White Plains, NY, United States; ^2^Weill Cornell Medicine, Feil Family Brain & Mind Research Institute, New York, NY, United States

**Keywords:** spinal cord injury, corticospinal, corticocortical, movement recovery, rehabilitation

## Abstract

Restoring sensory circuit function after spinal cord injury (SCI) is essential for recovery of movement, yet current interventions predominantly target motor pathways. Integrated cortical sensorimotor networks, disrupted by SCI, are critical for perceiving, shaping, and executing movement. Corticocortical connections between primary sensory (S1) and motor (M1) cortices are critical loci of functional plasticity in response to learning and injury. Following SCI, in the motor cortex, corticocortical circuits undergo dynamic remodeling; however, it remains unknown how rehabilitation shapes the plasticity of S1-M1 networks or how these changes may impact recovery of movement.

## Introduction

Sensory circuits provide essential components for accurate movement, including texture discrimination, spatial awareness, object perception, and tactile feedback (Abraira and Ginty, 2013). Sensory inputs for goal-directed movements provide information about location, size, weight, and shape of an object; therefore, successful integration of sensory inputs is key for generating a motor plan to execute a given movement. Additionally, sensory feedback during motor performance is required to refine ongoing movements. Sensorimotor integration is disrupted in spinal cord injury (SCI) (Edwards et al., 2019) and the recovery of sensory function will be a critical aspect in the recovery of movement.

## Sensory Afferents and Movement Recovery

Proprioceptive feedback transmitted through the dorsal column-medial lemniscal system is essential for movement control in healthy and injury conditions (Pearson, 1995; Windhorst, 2007; Tuthill and Azim, 2018). Within the spinal cord, proprioceptive and mechanoreceptive circuits are known to remodel below the level of SCI, providing an alternative circuit for transmission of afferent information (Hollis et al., 2015; Granier et al., 2020). After a lateral hemisection of the thoracic spinal cord, mice show spontaneous recovery of ipsilesional hindlimb control, whereas transgenic mice lacking muscle spindle-mediated proprioceptive input fail to recover locomotor function (Takeoka et al., 2014). Level-specific ablation of proprioceptive neurons demonstrated that locomotor recovery depends upon afferent input from below, but not above, the lesion (Takeoka and Arber, 2019). Furthermore, ablation of proprioceptive afferents after spontaneous locomotor recovery leads to a deterioration of the regained activity, indicating that proprioception is indispensable for both driving functional recovery as well as for maintaining that recovered function (Takeoka and Arber, 2019). Sensory function is not simply a passive byproduct of motor rehabilitation; sensorimotor training on discrete tactile substrates can improve recovery of locomotor function and tactile sensitivity after SCI (Martinez et al., 2009). The reactivation of S1 responses to cutaneous stimulation correlates with tactile recovery (Martinez et al., 2009). These findings suggest that rehabilitation can improve sensory function as well as sensorimotor-dependent movement recovery after SCI. A detailed understanding of the circuit mechanisms of rehabilitation-dependent S1 cortical plasticity is not known and further studies are required to address this mechanism that could provide critical data for designing therapeutic strategies for the recovery of movement after SCI.

## Sensory Cortex Responses to Spinal Cord Injury

Representations of somatosensory responses in S1 are highly plastic in response to nervous system damage, sensory experience, and learning. Cortical reorganization is a complex phenomenon that has been associated with both improved functional recovery and aberrant phantom sensations (Moxon et al., 2014). Thus, the underlying cellular mechanisms of somatosensory map plasticity and its consequences for cortical processing are highly relevant for shaping appropriate recovery of function after injury. SCI disrupts afferent input to the central nervous system and results in the reorganization of cortical sensory representations, or maps (Kaas et al., 2008; Moxon et al., 2014). In non-human primates, complete unilateral lesion of the ascending dorsal columns deactivates hand representations in area 3b of contralateral cortex (Jain et al., 1997, 2008). This loss of afferent input results in tactile deficits in the deprived forelimb and impaired performance on a reach-to-grasp task (Qi et al., 2013). In contrast to the effects on fine motor control, dorsal column lesion does not significantly impair locomotion, indicating that cortical sensory processing is not necessary for gross motor movements (Kaas et al., 2008).

**FIGURE 1:**
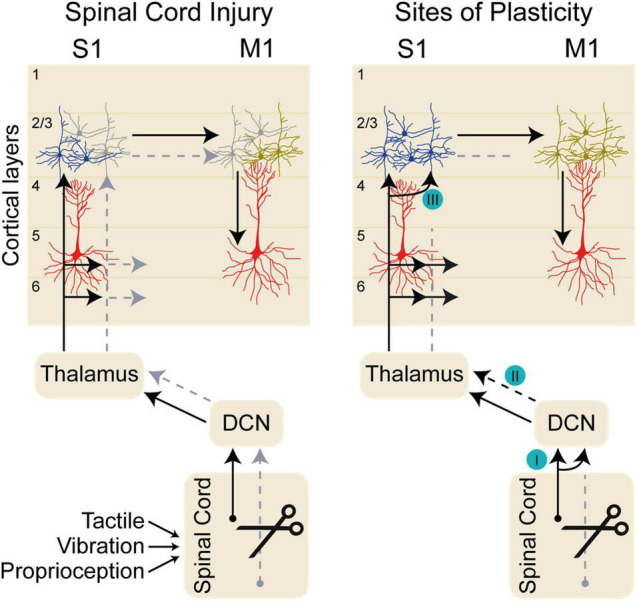
Dorsal column sensory pathways. The left diagram shows the pathway conveying tactile, proprioceptive, and vibratory sensory inputs through the dorsal column nuclei (DCN), to thalamus, and on to primary somatosensory cortex (S1) in normal conditions (black arrow). Sensory inputs arrive in S1 layers 2/3, 4, 5, and 6. Sensory information is transmitted between layer 2/3 neurons in S1 and primary motor cortex (M1), which in turn influences the output layer 5 neurons. Spinal cord injury impairs the sensory transmission along the sensory pathway (gray arrows) impacting the neurons in S1 and eventually in M1. The deafferented cortical neurons in S1 after spinal cord injury are shown in gray. The right diagram shows sites of axonal plasticity at distinct nuclei along the somatosensory pathway. (I) After spinal cord injury (SCI), inactivation of the cuneate dorsal column nuclei (DCN) eliminates aberrant face stimulation responses in the cortex (Kambi et al., 2014). (II) Reorganization of thalamic responses occurs after SCI (Jain et al., 2008); however, it is likely that the circuit plasticity supporting this functional reorganization arises in the DCN (Kambi et al., 2014). (III) Intracortical neurons within S1 drive local connectivity changes after SCI (Liao et al., 2016).

After incomplete lesions of the dorsal column, reactivation of S1 occurs with almost normal somatotopy (Yang et al., 2014; Qi et al., 2019). In contrast, complete dorsal column injuries immediately deactivate the hand representation in contralateral S1 area 3b (Jain et al., 1997, 2008). Over time, there is a reactivation of portions of the hand representation, likely mediated by sparse surviving primary afferents, second-order spinal neurons, and reorganization at each relay of somatosensory path: spinal cord, dorsal column nuclei (nucleus cuneatus), thalamus (ventral posterolateral nucleus, VPL), and cortex (S1) (Figure 1; Moxon et al., 2014). In chronic injury, this leads to afferent information from neighboring regions activating neurons in deafferented cortex and driving phantom sensations rather than functional recovery (Jain et al., 1997). Rehabilitation may be used to effectively shape S1 remodeling as fMRI imaging in non-human primates trained on a reach-to-grasp task shows reactivation of somatosensory cortex after unilateral dorsal column lesion S1 (Qi et al., 2021). In this study, cortex rendered unresponsive to vibrotactile stimulation by injury began to respond to stimuli as hand use improved. Thus, the balance between aberrant and beneficial S1 plasticity may be tilted through appropriate rehabilitation.

In rodents using extracellular electrophysiology recordings and functional magnetic resonance imaging (fMRI) it has been shown that electrical stimulation of the forelimb after thoracic SCI elicits responses in deafferented hindlimb S1 (Endo et al., 2007) and an expansion of forelimb S1 (Ghosh et al., 2009). Within S1, cortical responses to electrical hindpaw stimulation are eliminated in the hindpaw cortex and responses of forepaw recorded in the forepaw cortex are increased immediately after SCI (Aguilar et al., 2010; Humanes-Valera et al., 2013). This expansion of intact forelimb sensory responses into deafferented hindlimb S1 occurs as early as 3 days after injury and persists for several months (Endo et al., 2007). The initial expansion is similar to what has been observed in the motor system where evoked motor maps of intact regions above the level of injury expand and are strengthened in association with the loss of output from deafferented motor areas (Topka et al., 1991; Streletz et al., 1995; Mikulis et al., 2002; Nardone et al., 2013; Hollis et al., 2016). As in S1, targeted rehabilitation results in reorganization of M1 motor maps reorganize and functional recovery of movements.

## Anatomical Substrates Underlying S1 Remodeling

Neither the underlying neural architecture that supports S1 reorganization after SCI nor the extent of the reorganization has been clearly established. The reorganization of S1 after SCI appears to be less extensive than in M1. Forepaw S1 regions do not exhibit large-scale remodeling after SCI, but rather show a more limited expansion (Dutta et al., 2014). Whether this remodeling is limited by S1 circuitry is unknown. In M1, large-scale remodeling occurs after SCI and peripheral nerve injury, with early changes dependent on existing corticocortical circuitry (Huntley, 1997; Hollis et al., 2016).

In both rodents and non-human primates, there is evidence for anatomical reorganization of sensory circuits at the level of the brainstem after central and peripheral injuries. Cervical transection of dorsal roots (rhizotomy) in the rat has been shown to increase projections from *fasciculus gracilis* into the deafferented cuneate nucleus (Sengelaub et al., 1997). In primates, dorsal column injuries result in reorganization of afferent responses in thalamus and area 3b (Jain et al., 2008). Aberrant cortical representations appear to depend on changes in brainstem circuitry after SCI, as selective inactivation of the reorganized cuneate nucleus eliminates expansion of responses to face stimulation in area 3b (Figure 1I; Kambi et al., 2014).

For structural remodeling to provide a functional benefit, newly generated circuits must contribute to the function of the underlying sensory-motor networks. S1 plays a critical role in integrating and processing sensory inputs during motor learning. Similar to the activity in M1, S1 encodes muscle activity before movement initiation (Umeda et al., 2019). It appears that S1 receives pre-movement input from M1, while during movement S1 integrates M1 activity with afferent input (Umeda et al., 2019). The ability of S1 to influence synaptic plasticity in M1 relies on synapses in layer 2/3, which are a primary site for long term potentiation (LTP) within M1 (Kaneko et al., 1994a,b). The plasticity of sensory inputs to layer 2/3 M1 influences motor output through excitatory connections with deeper layer corticofugal neurons. *In vivo* intracellular recordings in cats have shown that LTP can be induced in M1 layer 2/3 (but not in deeper layers neurons) by high frequency stimulation in S1. This plasticity is a likely mechanism underlying motor learning (Keller et al., 1990) and rehabilitation from SCI.

In both S1 and primary motor (M1) cortex, layer 2/3 neurons are critical loci of functional plasticity in response to learning and injury (Figure 1III). Forelimb function relies on the sensory dorsal column-medial lemniscal circuit that carries tactile, vibration, and proprioceptive information to thalamic nucleus VPL before the circuit completes with projections to S1. In rodents, S1 is adjacent to M1 and is the primary source of corticocortical input to forelimb M1, indicating a major role for afferent feedback in shaping motor output (Colechio and Alloway, 2009). Within S1, layer 4, and to a lesser extent layers 2/3 and 5A, receive lemnisco-cortical input from VPL (Yamawaki et al., 2021). Sensory information spreads both vertically and horizontally throughout layer 2/3, suggesting that layer 2/3 S1 circuits integrate information from across diverse brain regions (Feldmeyer et al., 2013). Electrophysiology recordings in response to optogenetic activation in cortical slices have shown that layer 2/3 and 5A S1 corticocortical neurons excite layer 2/3 neurons in M1, with weaker connections to deeper layers (Yamawaki et al., 2021). Within M1, motor learning drives remodeling of both structure and activity of layer 2/3. Layer 2/3 neurons connect with deeper layer 5 corticofugal neurons, shaping cortical output. In M1, layer 2/3 excitatory neurons undergo a dynamic remodeling of network activity during task training, along with an increase in structural remodeling of dendritic spines (Peters et al., 2014). In a behavioral task in which trained forelimb movements on a joystick task were modified by external force affecting movement trajectory, mice made adaptive movements to counteract the effects of applied force (Mathis et al., 2017). Inhibition of S1 during the applied force abolished motor adaptation, demonstrating an active role for S1 processing of afferent input in modifying motor outputs (Mathis et al., 2017). The lateral corticocortical connections in layer 2/3 likely mediate S1-dependent motor adaptation needed for the appropriate modulation of movements. Plasticity of these layer 2/3 connections in both S1 and M1 may play a prominent role in functional recovery after SCI.

## Looking to the Future

The circuit mechanisms underlying functional reorganization of sensory and motor cortex after SCI are not well-characterized (Figure 1III). Studies in non-human primates and rodents that focused on reorganization of S1 after SCI have lacked the cellular resolution to measure the circuits involved. In part, this owes to a limitation of the techniques used: eliciting responses to artificial stimuli, rather than understanding endogenous responses to sensory stimuli; as well as large, meso-scale measures that cannot show real-time changes on a cellular level. Furthermore, most mapping studies have been limited by the use of anesthesia, and are not measures of active sensation in awake, behaving animals. The use of modern *in vivo* imaging tools and sensitive sensory and motor behavioral tasks (Morandell and Huber, 2017; Prsa et al., 2019) will facilitate a deeper understanding of active circuit changes following SCI.

## Author Contributions

YML and EH co-wrote the manuscript. Both authors contributed to the article and approved the submitted version.

## Conflict of Interest

The authors declare that the research was conducted in the absence of any commercial or financial relationships that could be construed as a potential conflict of interest.

## Publisher’s Note

All claims expressed in this article are solely those of the authors and do not necessarily represent those of their affiliated organizations, or those of the publisher, the editors and the reviewers. Any product that may be evaluated in this article, or claim that may be made by its manufacturer, is not guaranteed or endorsed by the publisher.
